# Complete Coding Sequences of One H9 and Three H7 Low-Pathogenic Influenza Viruses Circulating in Wild Birds in Belgium, 2009 to 2012

**DOI:** 10.1128/genomeA.00540-16

**Published:** 2016-06-09

**Authors:** Steven Van Borm, Toon Rosseel, Sylvie Marché, Mieke Steensels, Didier Vangeluwe, Annick Linden, Thierry van den Berg, Bénédicte Lambrecht

**Affiliations:** a Molecular Platform, Veterinary and Agrochemical Research Centre, Ukkel, Belgium; b Avian Virology and Immunology, Veterinary and Agrochemical Research Centre, Ukkel, Belgium; c Royal Belgian Institute of Natural Sciences (RBINS), Brussels, Belgium; d Faculty of Veterinary Medicine, University of Liège, Liège, Belgium

## Abstract

The complete coding sequences of four avian influenza A viruses (two H7N7, one H7N1, and one H9N2) circulating in wild waterfowl in Belgium from 2009 to 2012 were determined using Illumina sequencing. All viral genome segments represent viruses circulating in the Eurasian wild bird population.

## GENOME ANNOUNCEMENT

During the ongoing active surveillance for avian influenza (AI) in wild birds in Belgium from 2009 to 2012, several AI viruses were isolated from *Anseriformes* (1). We selected four viruses for whole-genome sequencing. Two H7N7 viruses, A/*Anas platyrhynchos*/Belgium/23852cls33/2012 (H7N7) and A/*Anas platyrhynchos*/Belgium/25576cls41/2012 (H7N7), were isolated from cloacal swabs taken from hunted mallards from different locations in southern Belgium (Sorée on 12 September 2012, and Grand-Leez on 6 November 2012, respectively). A/*Tadorna tadorna*/Belgium/3441_H160841/2009 (H7N1) was isolated from a cloacal swab taken from a common shelduck (*Tadorna tadorna*) caught for ringing in northern Belgium (Destelbergen on 25 March 2009). Last, A/*Anas platyrhynchos*/Belgium/24311pcs5/2012 (H9N2) was isolated from a cloacal swab from a mallard hunted in southern Belgium (Soheit-Tinlot on 9 September 2012).

Virus isolates (1 ml of allantoic fluid from first passage in embryonated chicken eggs) were pretreated by size-selective filtration (0.45 µM) and DNase treatment (100 U of DNase I [New England BioLabs] at 37°C for 1 h) before proceeding to total RNA extraction (QIAamp Viral RNA minikit; Qiagen). cDNA was produced using random hexamer primers and SuperScript III reverse transcriptase (Thermo Fisher Scientific), according to the manufacturer’s instructions. Sequencing libraries were prepared using 1 ng of cDNA and the Nextera XT kit (Illumina), according to the manufacturer’s instructions, quantified with the Kapa library quantification kit Illumina platforms (Kapa Biosystems), and fragment length distribution was verified using the Agilent Bioanalyzer with the high-sensitivity DNA kit (Agilent Technologies). Sequencing was performed using a MiSeq reagent kit version 3 (Illumina) with 2 × 300-bp paired-end sequencing. Eighteen libraries were multiplexed using standard Illumina indexing primers. The quality of the sequences was checked with the FastQC tool version 0.10.1 (http://www.bioinformatics.babraham.ac.uk/projects/fastqc/). Adapter sequences and stretches containing unidentified nucleotides (“N”) were trimmed using Cutadapt version 1.3 (2) prior to quality trimming using Sickle version 1.210 (*Q* score <30, length <50 bp) (3). *De novo* assembly was performed using Newbler version 2.9 (Roche). For each virus, 8 contigs were identified, corresponding to the 8 genomic RNA segments. When a contig did not correspond to the full-length segment sequence, a reference sequence (closest complete segment sequence in a BLASTN search) was selected and a reference assembly performed (Newbler version 2.9; Roche). Read assemblies were inspected using Tablet (4). Sequences were annotated using the NCBI Influenza Virus Sequence Annotation Tool (http://www.ncbi.nlm.nih.gov/genomes/FLU/Database/annotation.cgi). We obtained the complete coding sequences of the H7N1 and the H9N2 viruses, while minor parts of the extremities of the coding sequences of both H7N7 viruses were not obtained. The hemagglutinin cleavage site of all H7 viruses was determined to be low pathogenic (PEIPKGRGLF). The H9N2 virus presented hemagglutinin cleavage site amino acid sequence PAASNRGLF. Based on a BLASTn search (http://blast.ncbi.nlm.nih.gov/Blast.cgi [5]), all viral genome segments represent viruses circulating in the Eurasian wild bird population, confirming the previously documented dynamic nature of influenza viruses in their natural host and the important role of migratory birds in influenza virus ecology.

### Nucleotide sequence accession numbers.

The complete coding sequences of the viral RNA segments characterized in this study were assigned DDBJ/EMBL/GenBank accession numbers KU646957 to KU646988.
